# Bioactive Cembranoids from the Soft Coral Genus *Sinularia* sp. in Borneo

**DOI:** 10.3390/md16040099

**Published:** 2018-03-21

**Authors:** Takashi Kamada, Min-Cheol Kang, Chin-Soon Phan, Intan Irna Zanil, You-Jin Jeon, Charles S. Vairappan

**Affiliations:** 1 Laboratory of Natural Products Chemistry, Institute for Tropical Biology and Conservation, Universiti Malaysia Sabah, 88400 Kota Kinabalu, Sabah, Malaysia; takashi.kamada@ums.edu.my (T.K.); yuna123@hotmail.my (C.-S.P.); intanirnazanil@gmail.com (I.I.Z.); 2 Department of Marine Life Sciences, Jeju National University, Jeju 690-756, Korea; networksun@naver.com

**Keywords:** cembrane, terpenoid, soft coral, *Sinularia*, anti-inflammation, apoptosis, structure-activity relationship

## Abstract

Soft corals are known to be prolific producers of a wide spectrum of biologically active cembranoids. One new cembranoid, sinularolide F (**2**), along with three known compounds, cembranolide (**1**), (*E*,*E*,*E*)-6,10,14-trimethyl-3-methylene-*cis*-3α,4,5,8,9,12,13,15α-octahydrocyclo tetradeca[β]furan-2(*3H*)-one (**3**), and denticulatolide (**4**), were isolated from the Bornean soft coral *Sinularia* sp. Compounds **2** and **4** showed potential anti-inflammatory activities against lipopolysaccharide-stimulated RAW 264.7 with IC_50_ values less than 6.25 µg/mL and anticancer activity against HL60 cell lines. The compounds’ mechanisms of action were investigated via the Western blot evaluation of their protein markers. These activities could be attributed to the presence of tertiary methyl at C-8 and the compounds’ 3D configurations.

## 1. Introduction

**Figure 1:**
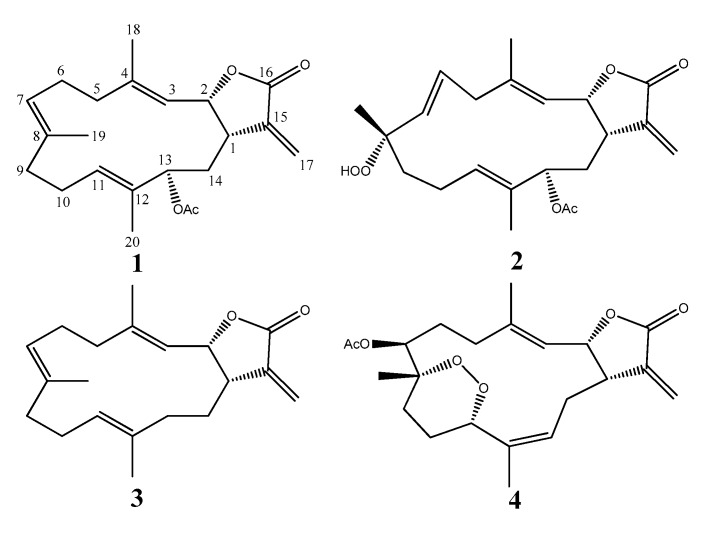
Structure of compounds **1**–**4**.

Our previous studies on the chemical constituents from Malaysian soft corals have yielded a variety of interesting metabolites, including sesquiterpenes [1,2,3,4], norsesquiterpenes [5], lobane [6], cembrane diterpenes [7,8,9,10,11], xenicanes [12,13], and sterols [4,14] with potent biological activities such as anti-inflammatory [1], antibacterial [3,4,6,9,10,13], and antifungal actions [11,12], as well as cytotoxicity against adult T-cell leukemia [11,12,13] and other cell lines [4,9]. Cembranes are 14-membered ring diterpenes that have frequently been reported as a major chemical constituent in soft corals of the order Alcyonacea [15,16,17,18]. Our continuous effort to better understand the chemical diversity of the soft coral *Sinularia* species in Bornean waters has led to the isolation of one new cembranoid, sinularolide F (**2**), along with three known compounds, cembranolide (**1**) [16], (*E*,*E*,*E*)-6,10,14-trimethyl-3-methylene-*cis*-3α,4,5,8,9,12,13,15α-octahydro-cyclotetradeca[β]furan-2(*3H*)-one (**3**) [15], and denticulatolide (**4**) [17,18], as shown in Figure 1. These compounds were tested for their anti-inflammatory activities in lipopolysaccharide (LPS)-activated RAW 264.7 macrophages and apoptosis activity against HL-60 cells. Based on the available literature, cembranolide (**1**) was reported (abstract from a conference paper) at the 27th Symposium on the Chemistry of Terpenes, Essential Oils and Aromatics (Kanazawa, Japan), which cannot be accessed from an internet source. It was reported unnamed, and its stereochemistry at C-13 as well as ^13^C-NMR data were not previously published [16,19]. In this regard, the relative configuration at C-13 in **1** is reported herein for the first time. Hence, this paper reports on the isolation, structure, and anti-inflammatory and apoptosis activities of the compound.

## 2. Results

### 2.1. Cembranolide *(**1**)*

**Figure 2:**
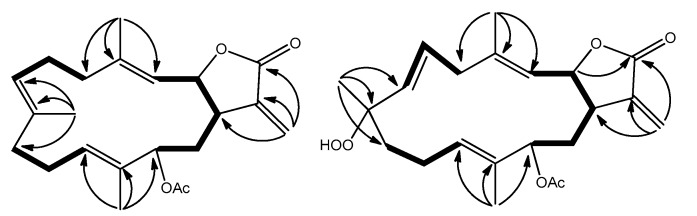
^1^H–^1^H COSY and key HMBC correlations of **1** and **2**.

Compound **1** was isolated as a colorless oil, with ${\left[\alpha \right]}_{D}^{25}$ +33.4 (*c* 0.50, CHCl_3_). The HRESI-MS of [M + Na]^+^ ion at *m*/*z* 381.2037, and NMR data (Table 1) established the molecular formula C_22_H_30_O_4_. The ^1^H- and ^13^C-NMR revealed one α-methylene-γ-lactone moiety at *δ*_H_ 6.25, 5.49, 5.53, and 3.10; *δ*_C_ 170.3, 138.3, 120.2, 77.6, and 40.2, an acetoxy group *δ*_H_ 2.05; *δ*_C_ 170.9 and 21.2, and three trisubstituted double bonds at *δ*_H_ 5.23, 4.90 and 4.70; *δ*_C_ 143.8, 133.2, 132.0, 129.8, 125.6 and 119.8. The planar structure was confirmed by the investigation of 2D NMR such as ^1^H–^1^H COSY and HMBC experiments (Figure 2). The HMBC correlations of H-13 to C-1 and C-21 suggested the presence of an acetoxymethine at C-13 (*δ*_H_ 5.04 (d, *J* = 11.0 Hz, 1H); *δ*_C_ 77.3).

**Figure 3:**
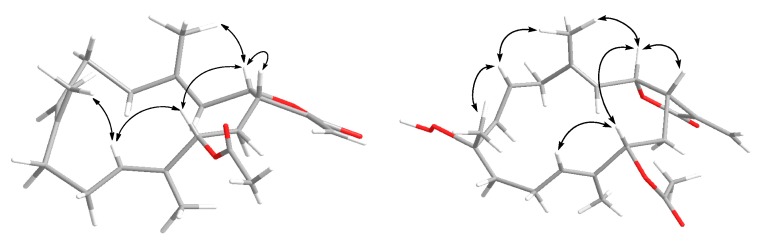
Key NOE correlations of **1** and **2**.

The relative stereochemistry of **1** was deduced from the NOESY experiment as well as the ^13^C-NMR chemical shifts. The ^13^C-NMR chemical shifts of C-18 at *δ*_C_ 15.1, C-19 at *δ*_C_ 15.2, and C-20 at *δ*_C_ 10.2 suggested that all double bonds had the *E* configurations [20,21]. The NOESY correlations observed between H-3/H2-5, H-7/H2-9, and H-11/H-13 also supported this deduction. The *cis*-fused lactone ring at C-1/C-2 was evident from the significant NOE interaction observed between H-1/H-2 and the coupling constant of ^3^*J*_1,2_ = 8.3 Hz [21,22,23]. Other NOE cross-peaks of H-2/H-13 and H-2/H_3_-18 indicated α-orientation of the 13-OAc. Furthermore, the up-field shift of methyl carbon at C-20 (<11.0 ppm) in **1** and **2** due to the γ-gauche effect also supported this assignment of the H-13 configuration [24]. In this regard, a pair of C-13 epimers with oxygenated methine at C-13 possessed a downfield shifted methyl carbon at C-20 (>13.0 ppm) due to the γ-gauche effect from β-orientation of the 13-OAc, while the opposite orientation of 13-OAc had a chemical shift of less than 11.0 ppm [24]. Together with the other detailed NOE interactions illustrated in Figure 3, the above observations established the structure of **1**.

### 2.2. Sinularolide F *(**2**)*

Compound **2** was isolated as a colorless oil, with ${\left[\alpha \right]}_{D}^{25}$ −17.0 (*c* 0.50, CHCl_3_). HRESI-MS [M + Na]^+^ ion at *m/z* 413.1933 was consistent to a molecular formula of C_22_H_30_O_6_, implying eight degrees of unsaturation. The ^13^C-NMR and DEPT spectra (Table 1) revealed the presence of three pairs of olefins at δ_C_ 142.4, 136.4, 131.9, 130.5, 128.6, and 120.0, an oxymethine carbon at *δ*_C_ 76.8, and one oxygenated quaternary carbon at *δ*_C_ 84.4. The carbon signal resonances at *δ*_C_ 170.2, 138.1, 120.9, 77.1, and 39.1 inferred a α-methylene-γ-lactone unit that was confirmed by comparison of NMR data with those of **1**. In this context, the structure of **2** resembled **1** except for the replacement of trisubstituted olefin at C-7/C-8 in **1** by sp^3^ methylene at C-7 and hydroperoxyl moiety at C-8 in **2**.

The ^1^H-^1^H COSY correlations suggested three proton-proton consecutive spin systems, as shown in Figure 2. The HMBC correlations from H_3_-18 to C-3, C-4, and C-5; H_3_-19 to C-7, C-8, and C-9; and H_3_-20 to C-11, C-12, and C-13 established the complete planar structure of **2**. In the same way, the relative configuration was also confirmed by NOESY spectrum (Figure 3) as well as the ^13^C-NMR chemical shifts. The geometry of the two trisubstituted olefins at C-3/C-4 and C-11/C-12 were assigned as *E* on the basis of the chemical shift of the olefinic methyl carbons at *δ*_C_ 16.1 and 10.2 for positions 18 and 20, respectively, as well as the absence of NOE correlations between H-3/H_3_-18 and H-11/H_3_-20 [20,21]. The coupling constant of 16.5 Hz between H-6 and H-7 confirmed the presence of *E* configuration double bond at C-6/C-7 [25]. The relative configurations at C-1, C-2, and C-8 were assigned as identical to those of crassumolide F due to similar key NOE cross-peaks observed between H-1/H-2, H-2/H-13, H-2/H_3_-18, H-6/H_3_-18, H-6/H_3_-19, and H-11/H-13 [17]. Furthermore, these assignments were also supported based on the comparison of ^1^H- and ^13^C-NMR data, vicinal coupling constants, and molecular model analysis [17]. Thus, the relative structure of **2** was deduced as sinularolide F.

### 2.3. Anti-Inflammatory Activity

**Figure 4:**
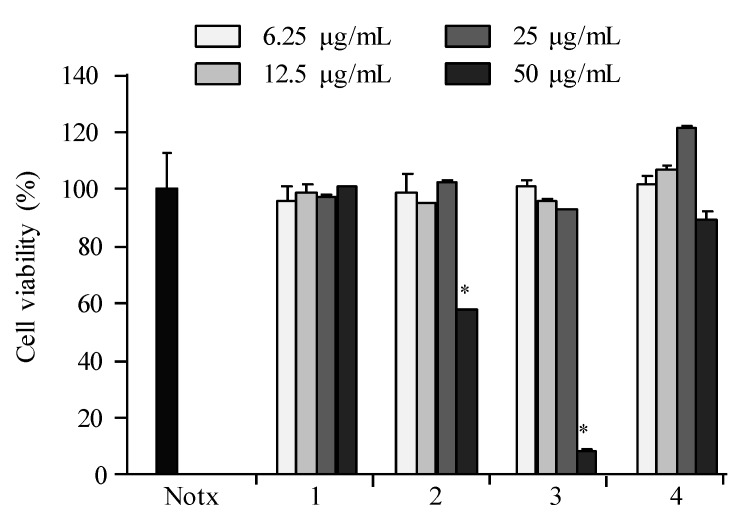
The effect of **1**–**4** on cell viability in RAW 264.7 macrophages. Cells were stimulated with LPS (1 μg/mL) in the presence of compounds (6.25, 12.5, 25, 50 μg/mL) for 24 h at 37 °C. The cell viability was assessed by MTT (3-(4,5-dimethylthiazole-2-yl)-2,5-diphenyltetrazolium bromide) assay. Values are expressed as means ± S.D. of triplicate experiments. * p < 0.05 indicates significant differences from the lipopolysaccharide (LPS)-stimulated group.

**Figure 5:**
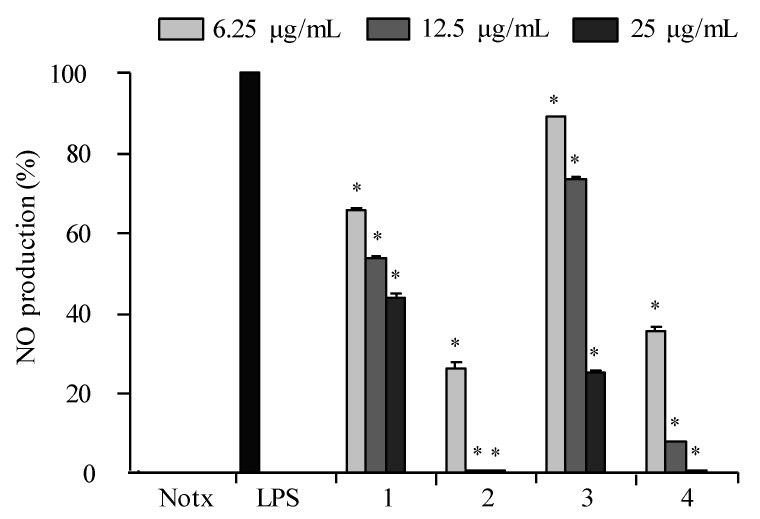
The effect of **1**–**4** on NO production in LPS-induced RAW 264.7 macrophages. Cells were stimulated with LPS (1 μg/mL) in the presence of compounds (6.25, 12.5, 25 μg/mL) for 24 h at 37 °C. Values are expressed as means ± S.D. of triplicate experiments. * p < 0.05 indicates significant differences from the LPS-stimulated group.

**Figure 6:**
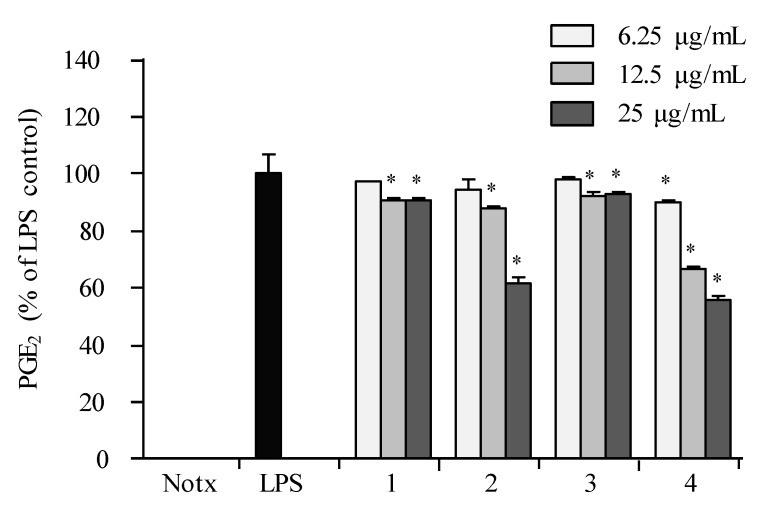
Inhibitory effect of compounds on the PGE_2_ production in RAW 264.7cells. Cells were stimulated with LPS (1 μg/mL) in the presence of compounds (6.25, 12.5, 25 μg/mL) for 24 h at 37 °C. Supernatantss were collected, and the PGE_2_ production in the supernatants were determined by ELISA. Values are expressed as means ± S.D. of triplicate experiments. * p < 0.05 indicates significant differences from the LPS-stimulated group.

**Figure 7:**
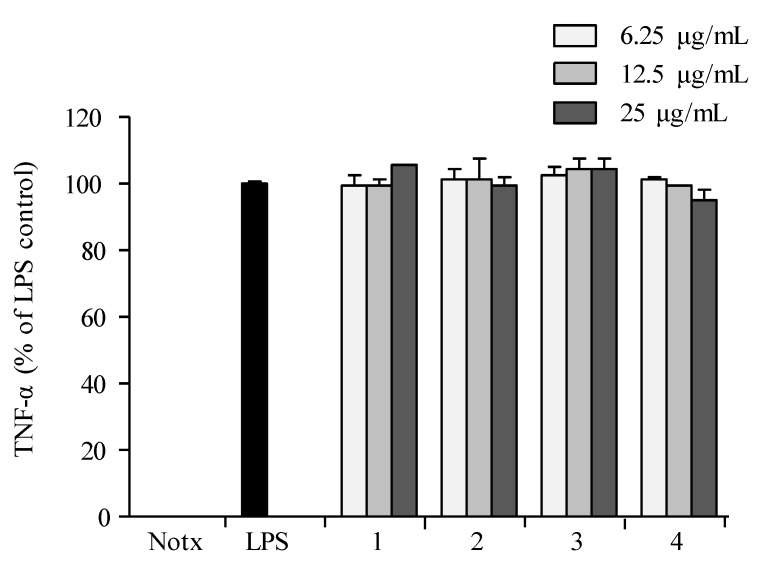
Inhibitory effect of compounds on the TNF-α production in RAW 264.7 cells. Cells were stimulated with LPS (1 μg/mL) in the presence of compounds (6.25, 12.5, 25 μg/mL) for 24 h at 37 °C. Supernatants were collected, and the TNF-α production in the supernatants were determined by ELISA. Values are expressed as means ± S.D. of triplicate experiments.

**Figure 8:**
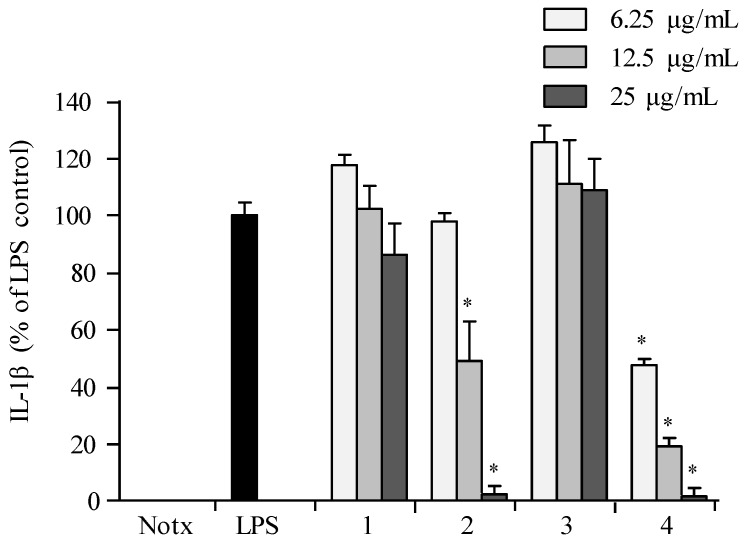
Inhibitory effect of compounds on the IL-1β production in RAW 264.7 cells. Cells were stimulated with LPS (1 μg/mL) in the presence of compounds (6.25, 12.5, 25 μg/mL) for 24 h at 37 °C. Supernatants were collected, and the IL-1β production in the supernatants were determined by ELISA. Values are expressed as means ± S.D. of triplicate experiments. * p < 0.05 indicates significant differences from the LPS-stimulated group.

**Figure 9:**
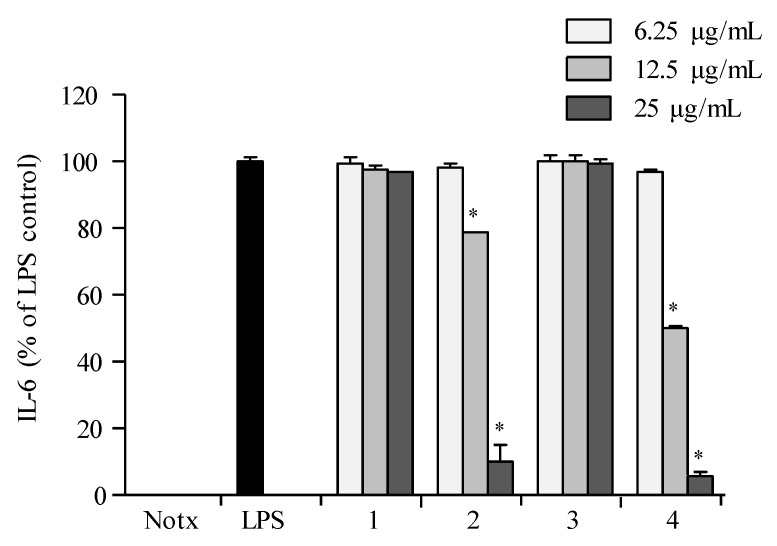
Inhibitory effect of compounds on the IL-6 production in RAW 264.7 cells. Cells were stimulated with LPS (1 μg/mL) in the presence of compounds (6.25, 12.5, 25 μg/mL) for 24 h at 37 °C. Supernatants were collected, and the IL-6 production in the supernatants were determined by ELISA. Values are expressed as means ± S.D. of triplicate experiments. * p < 0.05 indicates significant differences from the LPS-stimulated group.

The anti-inflammatory potential of compounds **1**–**4** was evaluated based on the accumulation of NO, PGE_2_, and pro-inflammatory cytokines (TNF-α, IL-1β, and IL-6) production as well as the pro-inflammatory protein (iNOS and COX-2) expression in LPS-induced RAW 264.7 macrophages. The results showed that **2** and **4** were the most active compounds, as shown in Figure 4, Figure 5, Figure 6, Figure 7, Figure 8 and Figure 9. The cell viability of **1**–**4** displayed no significant differences between the compound group (12.5, 25, and 50 μg/mL) and control group in the RAW 264.7 cells (Figure 4). The results indicated that concentrations of up to 25.0 µg/mL did not compromise the cell viability of RAW 264.7 macrophages. Therefore, a concentration of 25.0 µg/mL or less was used in further experiments.

The LPS treatment significantly elevated the production of NO as shown in Figure 5. The effects of **1**–**4** against the accumulation of NO production in LPS-stimulated RAW 264.7 cells showed that **2** and **4** were the most active against NO production at concentrations of 12.5 and 25.0 µg/mL as compared to that of negative control (LPS-induced RAW macrophages without the presence of the compound), as shown in Figure 5. The cell viability test proved that the inhibitory effect of **1**–**4** on NO production was not due to cytotoxic effects in RAW 264.7 cells. The data displayed that **2** and **4** exhibited inhibitions against PGE_2_ in LPS-induced RAW 264.7 macrophages in a dose-dependent manner (Figure 6).

Pre-treatment with **1**–**4** showed no significant inhibition against the accumulation of TNF-α production in LPS-treated RAW 264.7 cells when compared to those of the control group (Figure 7). Compounds **2** and **4** exhibited significant inhibition against the accumulation of interleukin (IL-1β and IL-6) productions in LPS-stimulated RAW 264.7 macrophages at a concentration of 25.0 µg/mL, with both interleukins’ production reduced to less than 10% in LPS-induced RAW 264.7 cells (Figure 8 and Figure 9).

**Figure 10:**
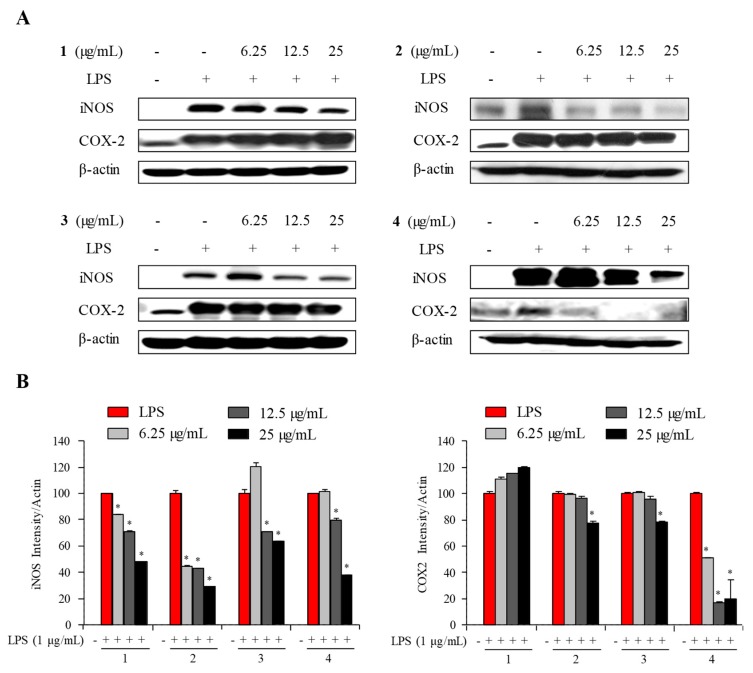
Inhibitory effect of compounds **1**–**4** on the protein level of iNOS and COX-2 in RAW 264.7 cells. Cells were treated for 1 h with LPS (1 μg/mL) alone or with LPS (1 μg/mL) coupled with different concentrations (6.25, 12.5, 25 μg/mL) of compounds **1**–**4**. Cell lysates were extracted, and protein levels of iNOS and COX-2 and β-actin were analyzed by Western blot.

To determine the mechanism by which the compound reduces LPS-stimulated NO and PGE_2_ production, the expression levels of iNOS and COX-2 in LPS-treated RAW 264.7 cells were monitored. The Western blot findings showed that the iNOS expression was inhibited in a concentration-dependent manner as observed in **2** and **4**, while **1** and **3** displayed little inhibition of iNOS and COX-2 expressions (Figure 10). These findings showed that **2** and **4** have abilities to inhibit NO, IL-1β, and IL-6 by down-regulating iNOS expression. In addition, they also displayed weak inhibition against PGE_2_ by slight suppression of COX-2 expression. These results indicated that compound **2** inhibits NO synthesis in RAW 264.7 cell by reducing the expression of iNOS protein. Additionally, these results indicated that compound **4** inhibits NO synthesis in RAW 264.7 cells by reducing the expression of both iNOS and COX-2 proteins. Therefore, compounds **2** and **4** may be promising iNOS inhibiting agents.

### 2.4. Apoptosis Activity

**Figure 11:**
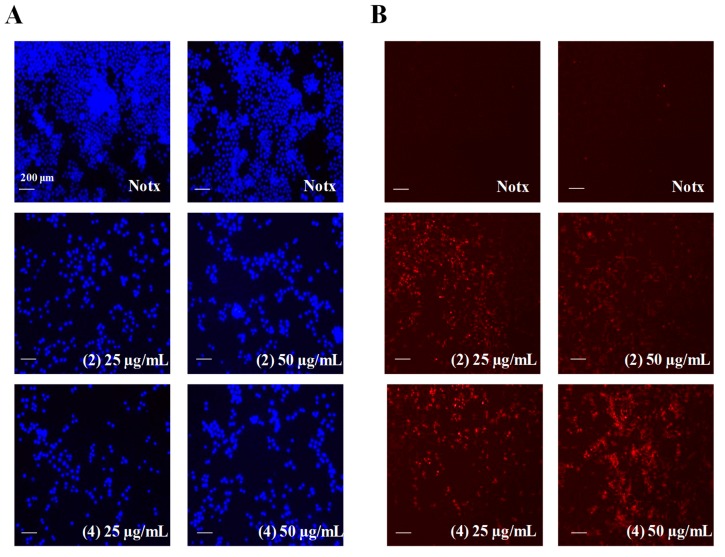
The effect of **2** and **4** on apoptotic body formation in HL-60 cells stained with (**A**) Hoechst 33342 and (**B**) propidium iodide.

Compounds **2** and **4** exhibited apoptosis activity against HL-60 cells during the screening process with cell viability below 30% at 25.0 µg/mL. Therefore, to examine the nuclear morphological changes in response to treatment of **2** and **4**, HL-60 cells were stained with the cell-permeable DNA dye Hoechst 33342 and visualized by fluorescence microscopy. The untreated cells have round intact nuclei as shown in Figure 11 (left). The results clearly show that **2** and **4** inhibited cell proliferation in a dose-dependent manner when compared to untreated cells. In addition, morphological characteristics such as chromatin condensation and bright nuclear fragmentation in the treated cells imply that **2** and **4** triggered apoptosis. Propidium iodide was used to confirm the presence of apoptotic HL-60 cells. Substantial numbers of stained cells were observed in treated cells compared to those of untreated cells, indicating the induction of apoptosis activity by **2** and **4**, as shown in Figure 11 (right).

**Figure 12:**
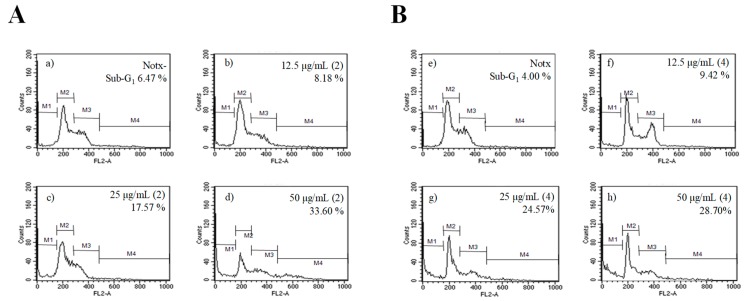
The effect of (**A**) **2** and (**B**) **4** on cell cycle pattern and apoptotic cell proportion in HL-60 cells. Apoptotic sub-G_1_ DNA content was detected by flow cytometry after propidium iodide staining. (**a**,**e**) control; (**b**) 12.5 µg/mL of **2**; (**c**) 25.0 µg/mL of **2**; (**d**) 50.0 µg/mL of **2**; (**f**) 12.5 µg/mL of **4**; (**g**) 25.0 µg/mL of **4**; (**h**) 50.0 µg/mL of **4**.

Flow cytometry analysis was carried out to quantify the apoptosis induction by **2** and **4**. The sub-G_1_ DNA content was 6.47% in control cells, while treatment of **2** displayed concentration-dependent increases in the proportion of apoptotic cells in sub-G_1_ populations of 8.18, 17.57, and 33.60% in 12.5, 25.0, and 50.0 µg/mL, respectively, as shown in Figure 12a–d. Meanwhile, dose-dependent increases in the proportion of apoptotic cells in sub-G_1_ populations upon exposure to **4** was 4.00 (control), 9.42, 24.57, and 28.70% with 12.5, 25.0, and 50.0 µg/mL, respectively, as shown in Figure 12e–h. This finding suggests that **2** and **4** might mediate their growth inhibitory effects on HL-60 cells by triggering an apoptosis event.

**Figure 13:**
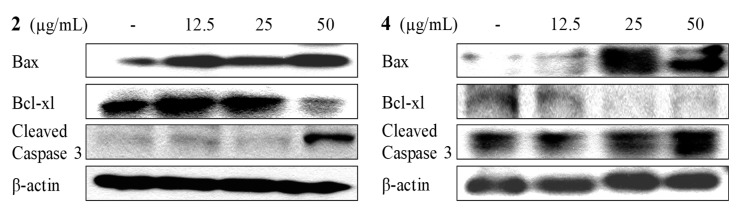
The effect of **2** and **4** on apoptotic-related protein expressions in HL-60 cells.

To determine the mechanism by which the compound activated apoptosis, the expressions of apoptotic proteins such as Bax, Bcl-xL, and caspase-3 were investigated. The results showed that treatment with **2** and **4** in HL-60 cells markedly increased the level of pro-apoptotic protein Bax and activated caspase-3, while expression of anti-apoptotic protein Bcl-xL was completely suppressed as shown in Figure 13. These findings suggested that **2** and **4** triggered the up-regulation of Bax, the down regulation of Bcl-xL, and the activation of caspase-3 in the apoptosis mechanism.

## 3. Discussion

The present investigation revealed the presence of one new cembranoid—sinularolide F (**2**)—along with three known cembrane-type compounds—cembranolide (**1**), (*E*,*E*,*E*)-6,10,14–trimethyl-3-methylene-*cis*-3a,4,5,8,9,12,13,15a-octahydrocyclotetradeca[*b*]furan-2(*3H*)-one (**3**), and denticulatolide (**4**)—in a population of Bornean *Sinularia* sp. collected at Mantanani Island, Sabah, Malaysia. The full assignment of **1** and **2** reported in this paper will serve as a future reference for these two compounds. In addition, compounds **2** and **4** possess anti-inflammatory activities by inhibiting NO, IL-1β, and IL-6 that are involved in down-regulating iNOS expression. In cellular function, Bax is a pro-apoptotic Bcl-2 protein which promotes apoptosis, while the caspase cascade was imperative in the execution of cell apoptosis [26]. Hence, metabolites **2** and **4** could suppress the proliferation of the HL-60 cancer cell line by apoptotic mechanisms that involved the up-regulation of Bax, the down-regulation of Bcl-xL, and the activation of caspase-3. From a structure–activity relationship perspective, these activities may be because **2** and **4** possess the β relative configuration of methyl at C-8 or both hydroperoxy and peroxy moieties favored the binding of anti-inflammatory and apoptotic response receptors [27]. Seven cembranoids—sarcrocrassocolides F–L with hydroxyl-bearing methyl or hydroperoxyl-bearing methyl at C-8—were reported as promising iNOS-inhibiting agents, but not against COX-2 expression; both the moiety and activities shared similarity to those of **2** [28]. Meanwhile, two cembranes—triangulenes A and B without hydroxyl-bearing methyl or hydroperoxyl-bearing methyl at C-8 but possessing α configuration methyl at C-8—showed no inhibition against iNOS expression [29]. This led to the assumption that their activities could be attributed to hydroxyl-bearing methyl or hydroperoxyl-bearing methyl and β configuration methyl at C-8. Therefore, compounds **2** and **4** can be promising candidates in our search for drugs for inflammatory diseases and human pro-myelocytic leukemia. The total synthesis and chemical modification of these two cembranoid scaffolds might create more potent lead pharmaceutical metabolites with these two properties.

## 4. Materials and Methods

### 4.1. General Experimental Procedures

The ^1^H-NMR (600 MHz) and ^13^C-NMR (150 MHz) spectra were recorded on a JEOL ECA 600 NMR (JEOL, Tokyo, Japan). The HRESIMS was acquired via LCMS-ESI-IT-TOF (Shimadzu, Kyoto, Japan). The AUTOPOL IV automatic polarimeter was used to measure the optical rotation (Rudolph Research Analytical, Hackettstown, NJ, USA). Infrared spectra were recorded on a Fourier transform infrared spectrometer (FTIR; Thermo Nicolet, Waltham, MA, USA). Pre-coated normal phase silica gel (Kieselgel 60 F_254_) thin-layer chromatography (TLC) glass plate and normal phase silica gel (Kieselgel 60, 70–230 mesh) column chromatography (Merck, Darmstadt, Germany).

### 4.2. Biological Material

Specimens of *Sinularia* sp. were collected from Mantanani Island, Sabah (6°44.015′ N, 116°19.202′ E) on the 18 April 2013. A voucher specimen (CSV-MI-3654) was deposited in the BORNEENSIS Collection of Institute for Tropical Biology and Conservation, Universiti Malaysia Sabah.

### 4.3. Extraction and Isolation

The fresh soft coral (780 g wet weight) was extracted in MeOH at room temperature for 5 days, subsequently filtered, concentrated, and partitioned between EtOAc/H_2_O, followed by partition between hexane/90% MeOH from EtOAc fraction. The hexane (1.2 g) fraction was subjected to column chromatography eluting with a gradient of hexane-EtOAc to obtain fractions 1–5. Fraction 2 (444.6 mg) was purified by preparative TLC using toluene to isolate **3** (100.2 mg). Purification from fraction 3 (379.6 mg) through repeated preparative TLC with CHCl_3_ and hexane–EtOAc (85:15) afforded **1** (30.4 mg), **2** (24.7 mg), and **4** (24.9 mg).

**1**: ${\left[\alpha \right]}_{D}^{25}$ +33.4 (*c* 0.50 CHCl_3_); IR (KBr) ν_max_ 1765 and 1730 cm^−1^; ^1^H- and ^13^C-NMR spectral data: see Table 1; HRESI-MS [M + Na]^+^ ion at *m*/*z* 381.2037 (calcd. for C_22_H_30_O_4_Na, 381.2036).

**2**: ${\left[\alpha \right]}_{D}^{25}$ −17.0 (*c* 0.50 CHCl_3_); IR (KBr) ν_max_ 3310, 1760 and 1731 cm^−1^; ^1^H- and ^13^C-NMR spectral data: see Table 1; HRESI-MS [M + Na]^+^ ion at *m*/*z* 413.1933 (calcd. for C_22_H_30_O_6_Na, 413.1935).

### 4.4. Anti-Inflammatory Assay

The murine macrophage cell line RAW 264.7 was purchased from the Korean Cell Line Bank (Seoul, Korea). The RAW 264.7 cell line was cultured in Dulbecco’s modified Eagle’s medium (DMEM; Thermo Fisher Scientific, Waltham, MA, USA) supplemented with 100 U mL^−1^ of penicillin (Thermo Fisher Scientific, Waltham, MA, USA), 100 μg mL^−1^ of streptomycin, and 10% fetal bovine serum (FBS; Thermo Fisher Scientific, Waltham, MA, USA) which was maintained in 5% CO_2_ at 37 °C. The cells were sub-cultured every two days, and exponential phase cells were used throughout the experiments. In the cytotoxicity assay, RAW 264.7 cells (1.0 × 10^5^ cells mL^−1^) were seeded in 96-well plate, followed by treatment of compounds at various concentrations and 3-(4,5-dimethylthiazole-2-yl)-2,5-diphenyltetrazolium bromide (MTT) stock solution. The quantity of formazan was measured at 540 nm by ELISA reader (Tecan Co. Ltd., Melbourne, Australia). The determination of nitric oxide (NO) and pro-inflammatory cytokines (TNF-α, IL-1β and IL-6) productions were carried out by pre-cultured RAW 264.7 cells (1.0 × 10^5^ cells mL^−1^) for 24 h, subsequently pre-incubated with compounds for 1 h, followed by treatment with LPS (1 μg mL^−1^) and incubated for 24 h. Griess reagent (1% sulfanilamide and 0.1% naphthylethylenediamine dihydrochloride in 2.5% phosphoric acid), the competitive enzyme immunoassay kit (R & D Systems, Minneapolis, MN, USA), and the ELISA kit (R & D Systems, Minneapolis, MN, USA) were used to quantify NO, TNF-α, IL-1β, and IL-6 productions.

Murine macrophage RAW 264.7 cells were pre-incubated for 18 h, then stimulated with LPS (1 μg/mL) in the presence of compounds for the indicated times. After incubation, the cells were collected and washed twice with cold PBS. The cells were lysed in a lysis buffer (50 mM Tris–HCl (pH 7.5), 150 mM NaCl, 1% Nonidet P-40, 2 mM EDTA, 1 mM EGTA, 1 mM NaVO_3_, 10 mM NaF, 1 mM dithiothreitol, 1 mM phenylmethylsulfonyl fluoride, 25 μg/mL aprotinin, and 25 μg/mL leupeptin) and maintained on ice for 30 min. The cell lysates were washed via centrifugation, and the protein concentrations were determined using a BCA™ protein assay kit (Thermo Fisher Scientific, Waltham, MA, USA). Aliquots of the lysates (30–50 μg of protein) were separated on 12% SDS-polyacrylamide gel and transferred onto a polyvinylidene fluoride (PVDF) membrane (BIO-RAD) with a glycine transfer buffer (192 mM glycine, 25 mM Tris–HCl (pH 8.8), 20% methanol (*v*/*v*)). After blocking the nonspecific site with 1% bovine serum albumin (BSA), the membrane was incubated overnight with specific primary antibody (anti-mouse iNOS (1:1000; Calbiochem, San Diego CA, USA), anti-mouse COX-2 (1:1000; BD Biosciences Pharmingen, San Diego, CA, USA) and β-actin) at 4 °C. The membrane was then incubated for an additional 60 min with a peroxidase-conjugated secondary antibody (1:5000, Vector Laboratories, Burlingame, CA, USA) at room temperature. The immunoactive proteins were detected using an enhanced chemiluminescence (ECL) Western blotting detection kit (Thermo Fisher Scientific, Waltham, MA, USA). The assay was conducted in accordance with known procedure [1,30,31].

### 4.5. Apoptotic Assay

The human cancer promyelocytic leukaemia (HL-60) cell line was cultured in Roswell Park Memorial Institute media (RPMI-1640; Thermo Fisher Scientific, Waltham, MA, USA) supplemented with 100 U mL^−1^ of penicillin, 100 μg mL^−1^ of streptomycin, and 10% heat-inactivated FBS which was maintained in 5% CO_2_ at 37 °C. The HL-60 cell line was cultured in RPMI supplemented with 10% heat-inactivated FBS at 5% CO_2_ for 37 °C. The cells were sub-cultured every three days. In the cell viability assay, HL-60 cells (1.0 × 10^5^ cells mL^−1^) were seeded in a 96-well plate, followed by treatment of compounds at various concentrations and MTT stock solution. The quantity of formazan was measured at 540 nm by ELISA reader (Tecan Co. Ltd., Melbourne, Australia). The nuclear morphology study was carried out by seeding HL-60 cells (1.0 × 10^5^ cells mL^−1^) for 16 h. Subsequently, compounds were added, followed by incubation for 12 h. The nuclear morphology of the cells was evaluated using the cell-permeable DNA dye Hoechst 33342 and propidium iodide (PI). Cells with homogeneously-stained nuclei were considered viable, whereas the presence of chromatin condensation and/or fragmentation was indicative of apoptosis. The cells were placed in 24-well plates at a concentration of 1 × 10^5^ cells mL^−1^ (950 μL). Sixteen hours after plating, the cells were treated with various concentrations of the compounds (50 μL). After 16 h, 3 μL of Hoechst 33342 (stock 10 mg mL^−1^) and PI (stock 10 mg mL^−1^) were added to each well, followed by 10 min of incubation at 37 °C. The stained cells were then observed under a fluorescence microscope equipped with a CoolSNAP-Pro color digital camera (Carsen Group, Markham, ON, Canada), in order to examine the degree of nuclear condensation. HL-60 cells (4 × 10^5^ cells/mL) were treated with 150 μM of ACAN for various time points and harvested. The cells were lysed in a lysis buffer (50 mM Tris–HCl (pH 7.5), 150 mM NaCl, 1% Nonidet P-40, 2 mM EDTA, 1 mM EGTA, 1 mM NaVO_3_, 10 mM NaF, 1 mM dithiothreitol, 1 mM phenylmethylsulfonyl fluoride, 25 μg/mL aprotinin, and 25 μg/mL leupeptin) and kept on ice for 30 min. Antibodies against Bax, Bcl-2, caspase-9, and β-actin were purchased from Cell Signaling Technology (Bedford, MA, USA). Cell lysates were washed by centrifugation, and protein concentrations were determined using the BCA^TM^ protein assay kit (Thermo Fisher Scientific, Waltham, MA, USA) Aliquots of the lysates (30 μg of protein) were separated on a 12% SDS–polyacrylamide gel and transferred onto a polyvinylidene fluoride membrane (BIO-RAD) with a glycine transfer buffer (192 mM glycine, 25 mM Tris–HCl (pH 8.8), and 20% methanol (*v/v*)). After the nonspecific site was blocked with 1% bovine serum albumin, the membrane was incubated with specific primary antibody (1:1000) at 4 °C overnight. The membrane was further incubated for 60 min with a peroxidase-conjugated secondary antibody (1:5000; Vector Laboratories, Burlingame, CA, USA) at room temperature.The resulting bands were visualized on X-ray film using ECL detection reagent (Amersham Biosciences, Piscataway, NJ, USA). The assay was conducted in accordance with known procedure [32].

### 4.6. Statistical Analysis

Data were analyzed using the Statistical Package for the Social Sciences (SPSS) package for Windows (Version 8). Values were expressed as means ± standard error (SE). A *p*-value of less than 0.05 was considered significant.

## Figures and Tables

**Table 1:** ^13^C- and ^1^H-NMR data (150 and 600 MHz in CDCl_3_; *δ* in ppm, *J* in Hz) of **1** and **2**.

No.	1		2	
	*δ*_C_	*δ*_H_	*δ*_C_	*δ*_H_
1	40.2 (CH)	3.10 ddd (3.4, 8.3, 11.7)	39.1 (CH)	3.12 ddd (2.8, 8.3, 11.7)
2	77.6 (CH)	5.53 dd (8.3, 11.0)	77.1 (CH)	5.48 dd (8.3, 11.0)
3	119.8 (CH)	4.90 d (11.0)	120.0 (CH)	5.00 d (11.0)
4	143.8 (C)		142.4 (C)	
5	39.6 (CH_2_)	2.16 m	43.1 (CH_2_)	2.78 m
		1.80 dt (4.8, 12.4)		2.76 m
6	24.2 (CH_2_)	2.39 m	128.6 (CH)	5.80 td (8.3, 15.8)
		2.03 m		
7	125.6 (CH)	4.70 br d (8.9)	136.4 (CH)	5.46 d (15.8)
8	133.2 (C)		84.4 (C)	
9	39.5 (CH_2_)	2.31 br d (13.8)	37.7 (CH_2_)	1.77 m
		2.14 m		1.72 m
10	22.9 (CH_2_)	2.14 m	22.5 (CH_2_)	2.36 m
		2.00 m		1.96 m
11	129.8 (CH)	5.23 dd (5.5, 9.6)	130.5 (C)	5.31 dd (4.1, 10.3)
12	132.0 (C)		131.9 (C)	
13	77.3 (CH)	5.04 d (11.0)	76.8 (CH)	5.00 d (11.7)
14	32.4 (CH_2_)	2.41 m	32.6 (CH_2_)	2.20 ddd (2.8, 11.7, 14.4)
		1.54 m		1.52 dd (11.7, 14.4)
15	138.3 (C)		138.1 (C)	
16	170.3 (C)		170.2 (C)	
17	120.2 (CH_2_)	6.25 d (3.4)	120.9 (CH_2_)	6.28 d (3.4)
		5.49 d (3.4)		5.52 d (3.4)
18	15.1 (CH_3_)	1.75 s	16.1 (CH_3_)	1.91 s
19	15.2 (CH_3_)	1.58 s	20.7 (CH_3_)	1.42 s
20	10.2 (CH_3_)	1.60 s	10.2 (CH_3_)	1.61 s
13-OAc	170.9 (C)		170.9 (C)	
	21.2 (CH_3_)	2.05 s	21.2 (CH_3_)	2.05 s

## Supplementary Materials

The following are available online at http://www.mdpi.com/1660-3397/16/4/99/s1. Figure S1: ^1^H-NMR spectrum of **1** in CDCl_3_ (600 MHz); Figure S2: ^13^C-NMR spectrum of **1** in CDCl_3_ (150 MHz); Figure S3: HSQC spectrum of **1** in CDCl_3_; Figure S4: ^1^H-^1^H COSY spectrum of **1** in CDCl_3_; Figure S5: HMBC spectrum of **1** in CDCl_3_; Figure S6: NOESY spectrum of **1** in CDCl_3_; Figure S7: HRESI-MS data of **1**; Figure S8: ^1^H-NMR spectrum of **2** in CDCl_3_ (600 MHz); Figure S9: ^13^C-NMR spectrum of **2** in CDCl_3_ (150 MHz); Figure S10: HSQC spectrum of **2** in CDCl_3_; Figure S11: ^1^H-^1^H COSY spectrum of **2** in CDCl_3_; Figure S12: HMBC spectrum of **2** in CDCl_3_; Figure S13: NOESY spectrum of **2** in CDCl_3_; Figure S14: HRESI-MS data of **2**.
