# Supplementary material for: Bioactive Cembranoids from the Soft Coral Genus Sinularia sp. in Borneo
Source: Mar Drugs. 2018 Mar 21;16(4):99. doi: 10.3390/md16040099 (PMC5923386; doi:10.3390/md16040099)
Supplement: Supplementary file 1 [file marinedrugs-16-00099-s001.pdf]

Supplementary Materials

Figure S1. ^{1}H-NMR spectrum of **1** in $CDCl_3$ (600 MHz).
Figure S2. ^{13}C-NMR spectrum of **1** in $CDCl_3$ (150 MHz).
Figure S3. HSQC spectrum of **1** in $CDCl_3$.
Figure S4. ^{1}H-^{1}H COSY spectrum of **1** in $CDCl_3$.
Figure S5. HMBC spectrum of **1** in $CDCl_3$.
Figure S6. NOESY spectrum of **1** in $CDCl_3$.
Figure S7. HRESI-MS data of **1** .
Figure S8. ^{1}H-NMR spectrum of **2** in $CDCl_3$ (600 MHz).
Figure S9. ^{13}C-NMR spectrum of **2** in $CDCl_3$ (150 MHz).
Figure S10. HSQC spectrum of **2** in $CDCl_3$.
Figure S11. ^{1}H-^{1}H COSY spectrum of **2** in $CDCl_3$.
Figure S12. HMBC spectrum of **2** in $CDCl_3$.
Figure S13. NOESY spectrum of **2** in $CDCl_3$.
Figure S14. HRESI-MS data of **2**.

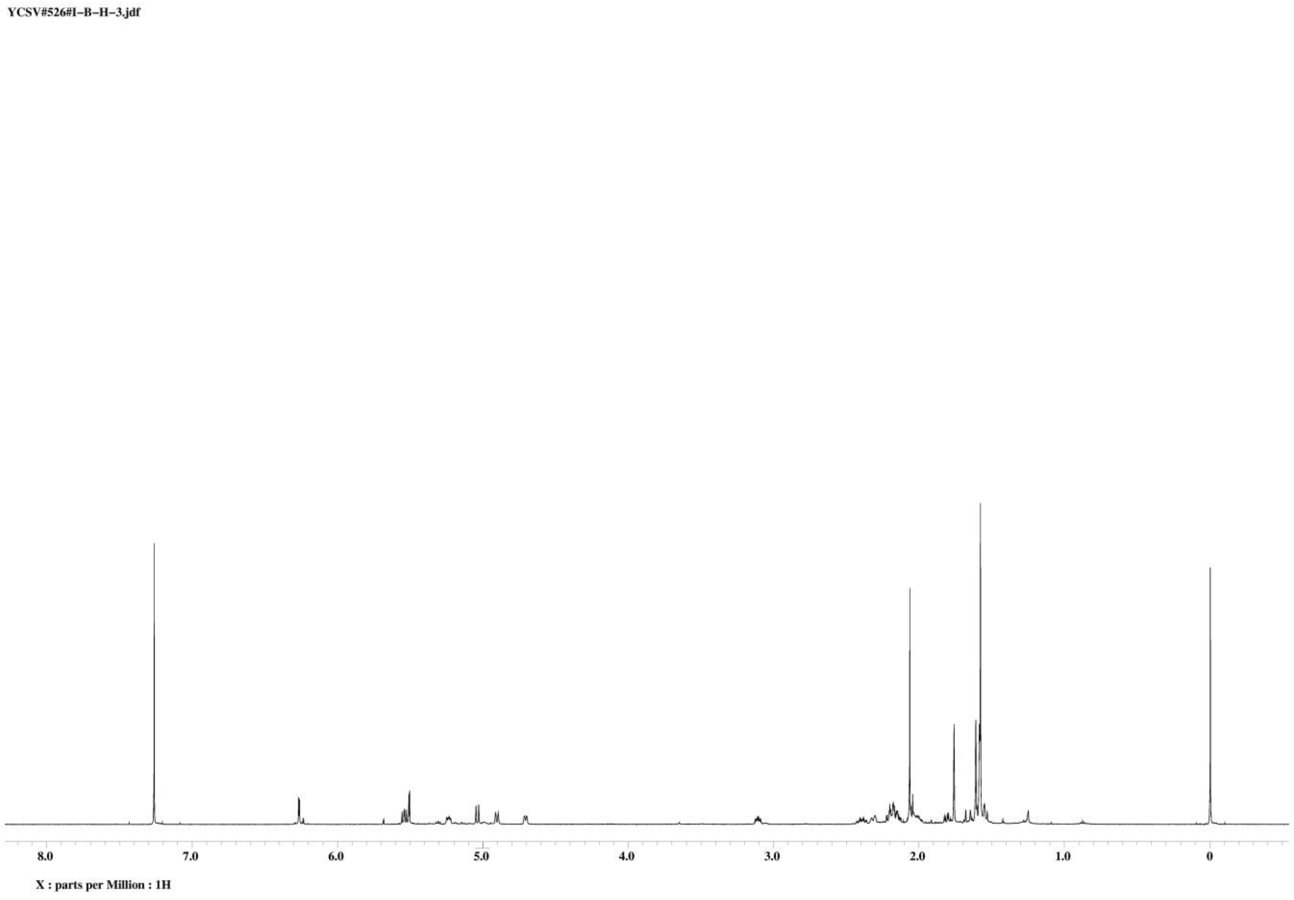

Figure S1. ^{1}H-NMR spectrum of **1** in $CDCl_3$ (600 MHz).

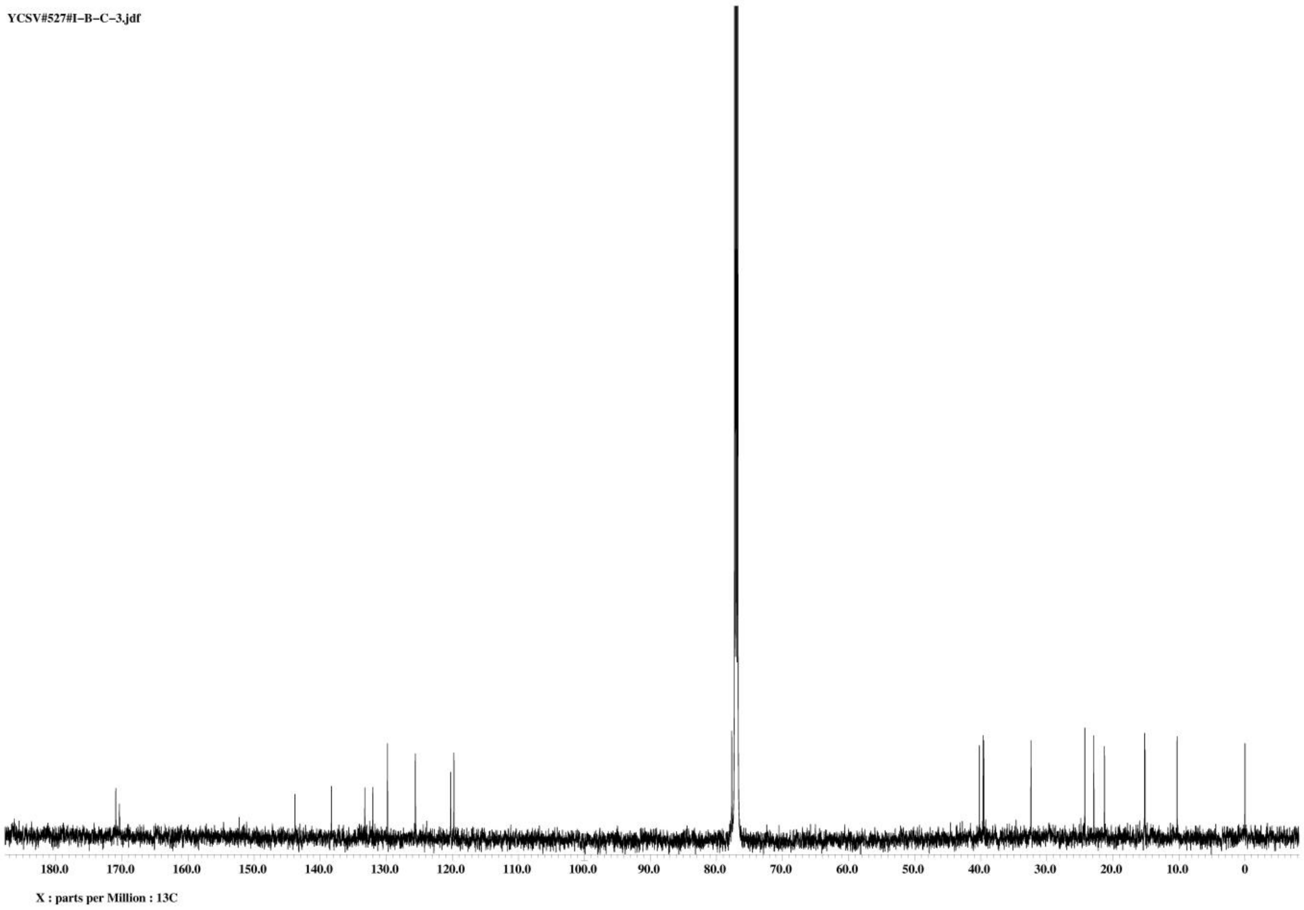

Figure S2. ^{13}C-NMR spectrum of **1** in $CDCl_3$ (150 MHz).

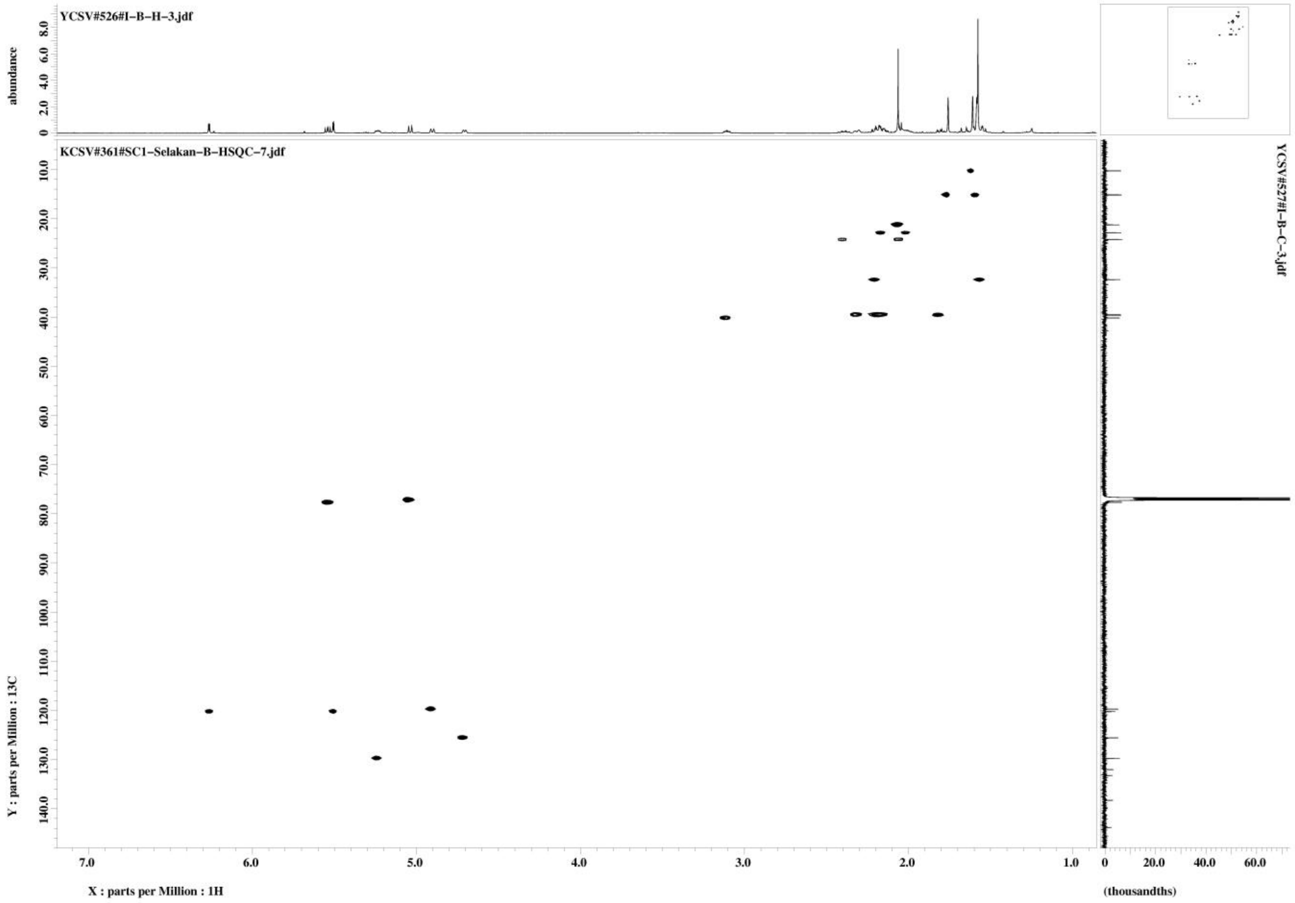

Figure S3. HSQC spectrum of **1** in $CDCl_3$

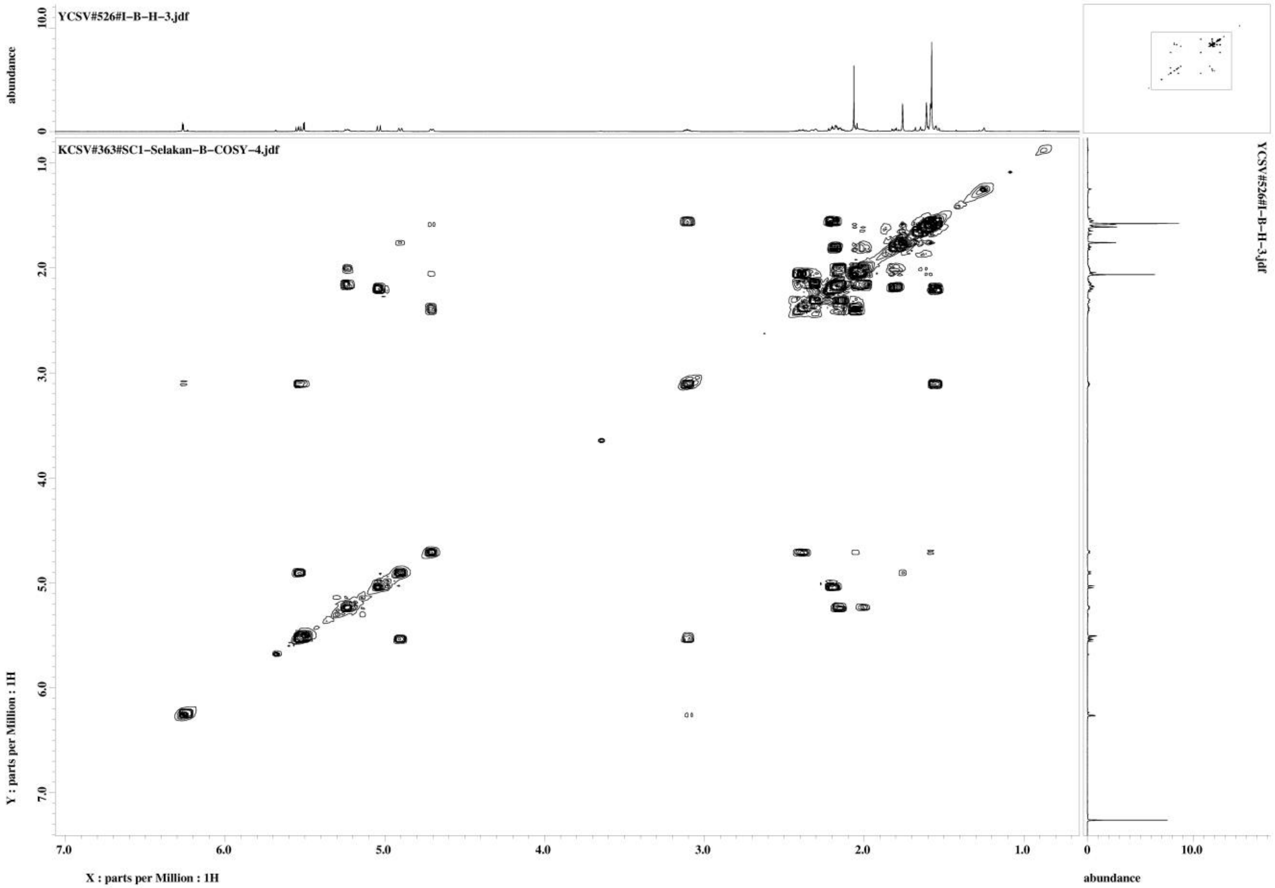

Figure S4. ^{1}H-^{1}H COSY spectrum of **1** in $CDCl_3$.

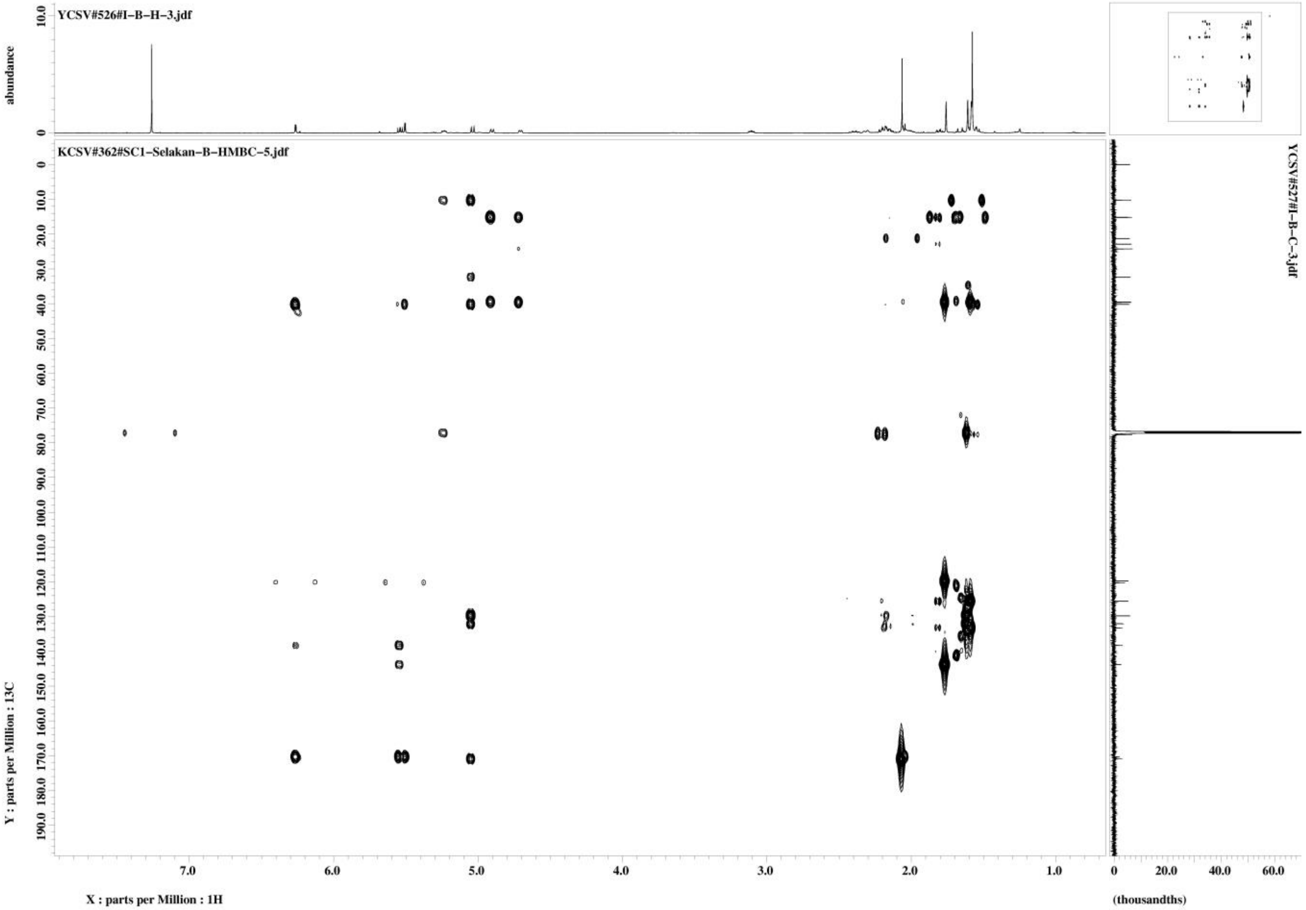

Figure S5. HMBC spectrum of **1** in $CDCl_3$.

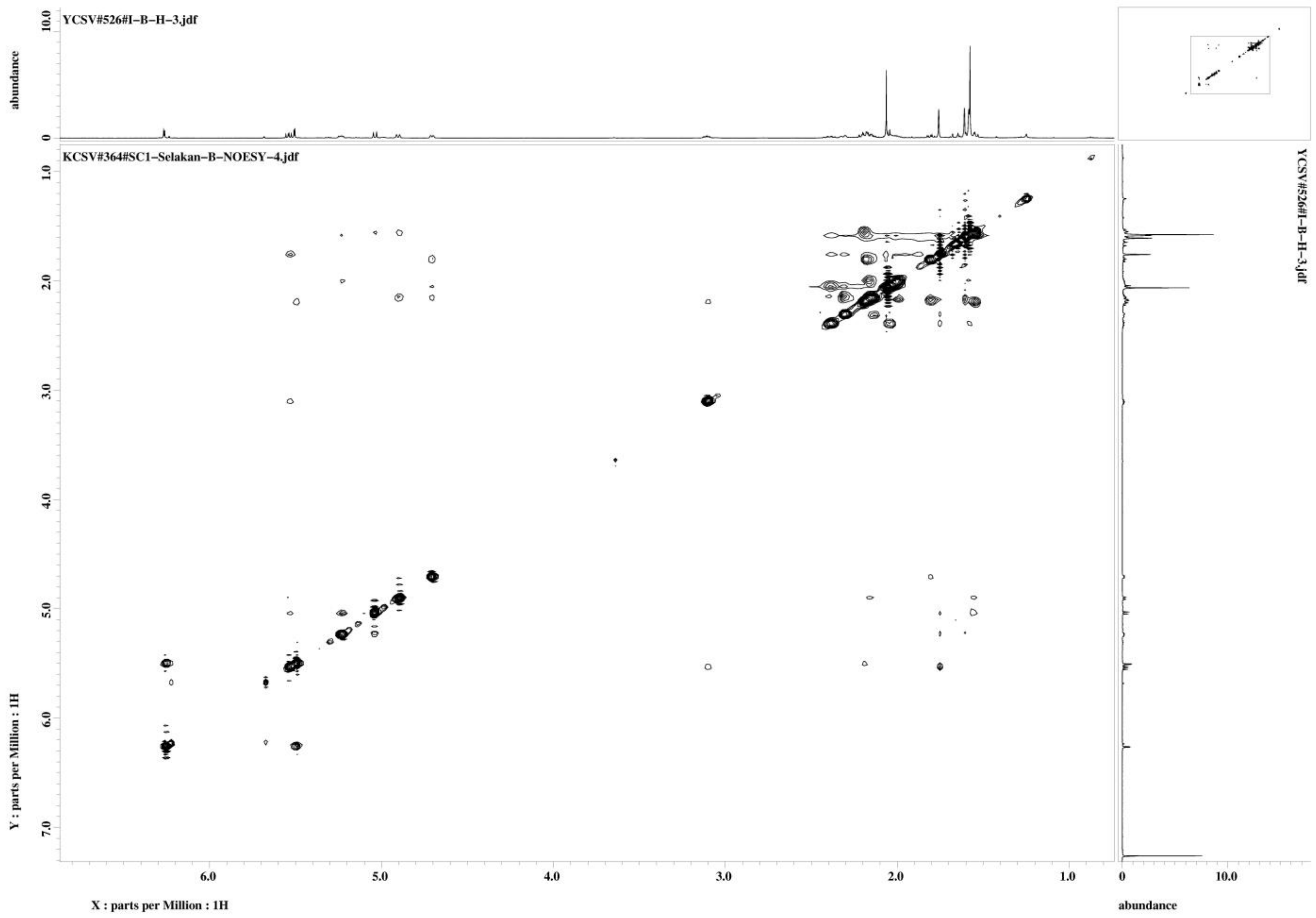

Figure S6. NOESY spectrum of **1** in $CDCl_3$.

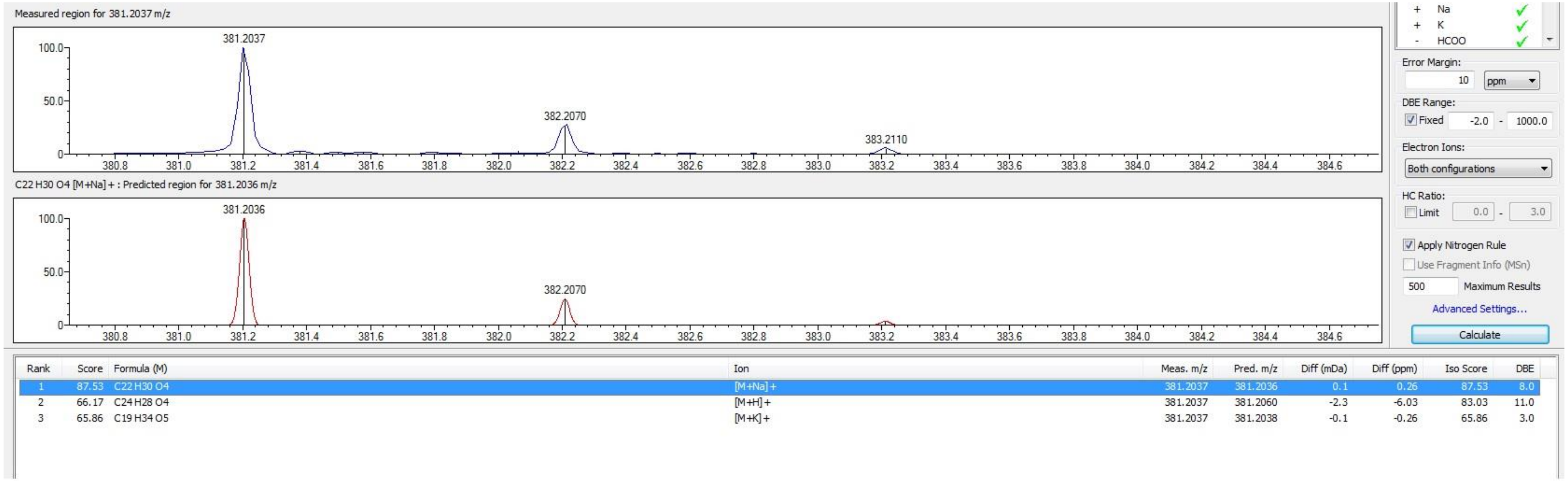

Figure S7. HRESI-MS data of **1**.

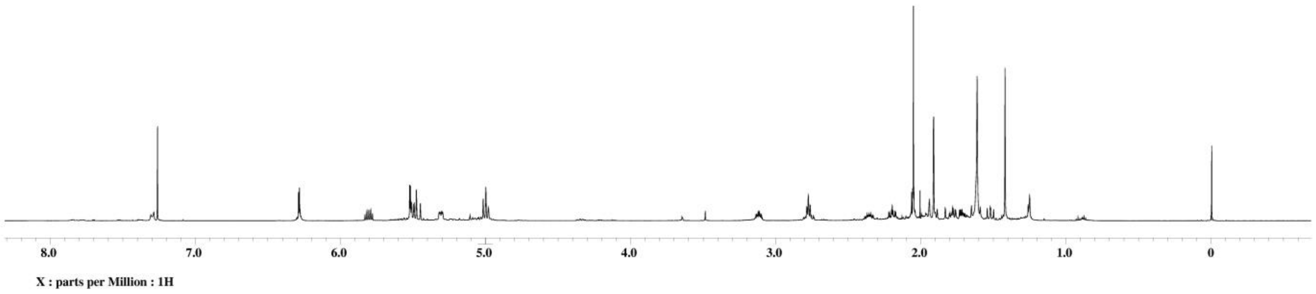

Figure S8. ^{1}H-NMR spectrum of **2** in $CDCl_3$ (600 MHz).

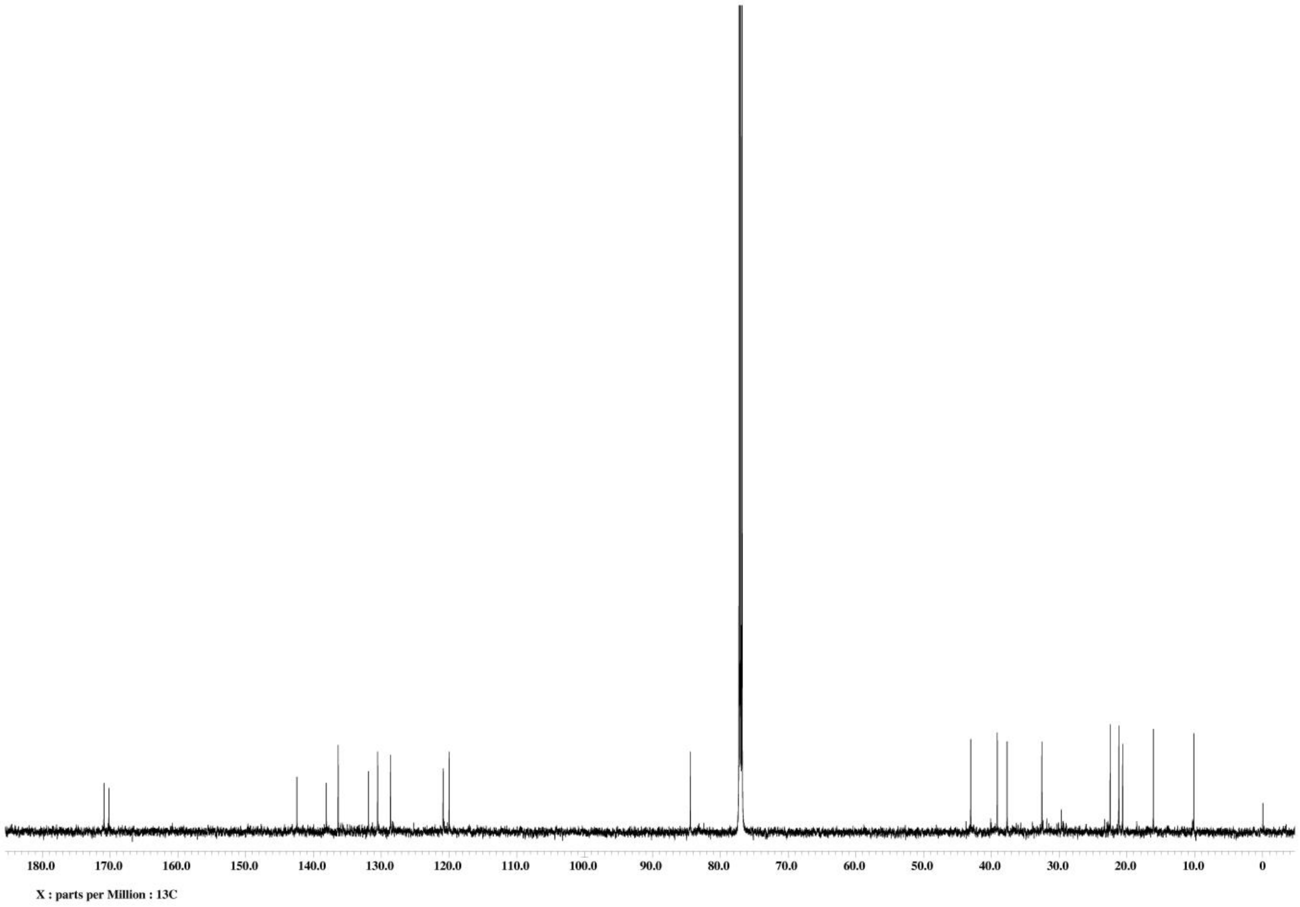

Figure S9. ^{13}C-NMR spectrum of **2** in $CDCl_3$ (150 MHz).

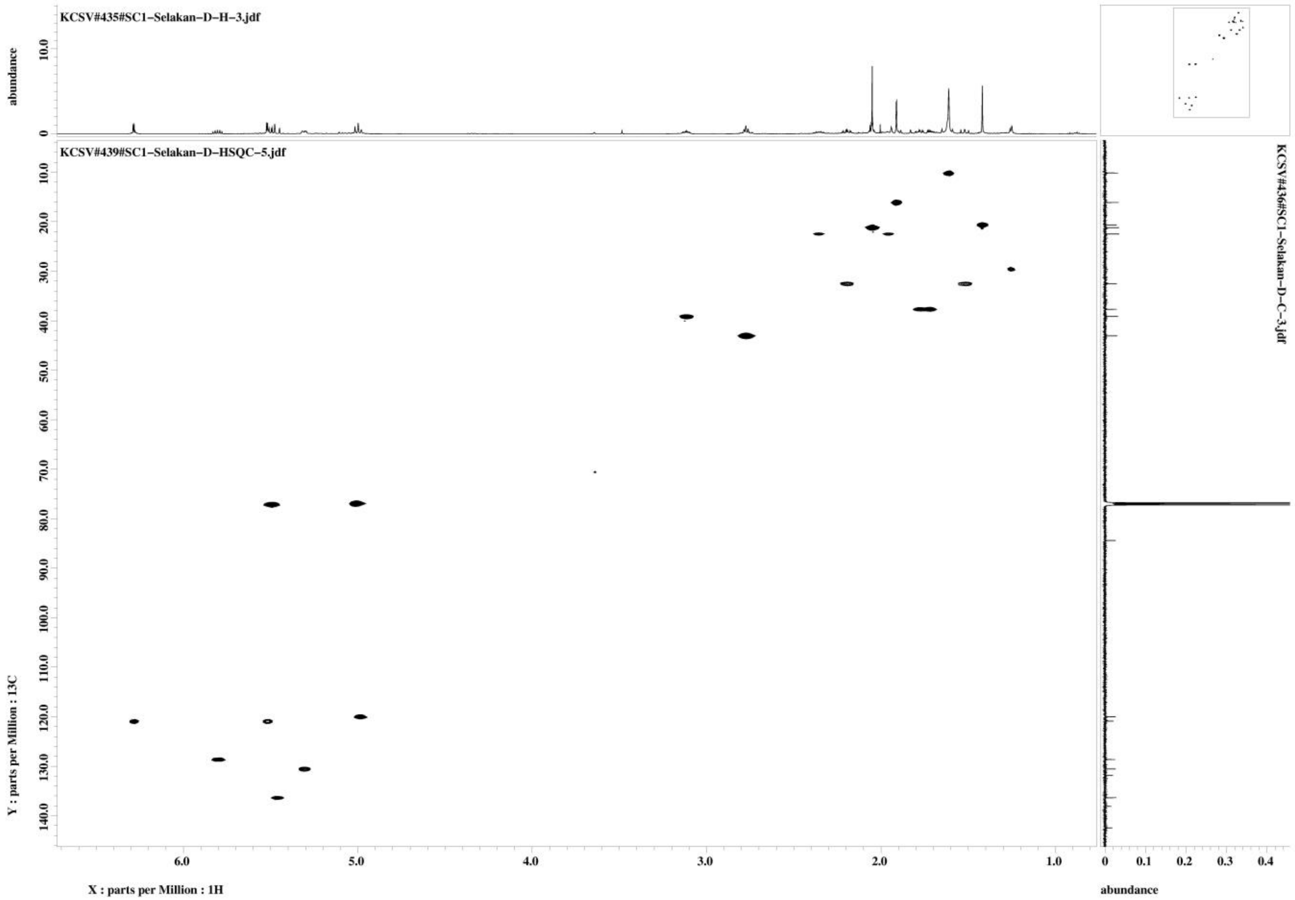

Figure S10. HSQC spectrum of **2** in $CDCl_3$.

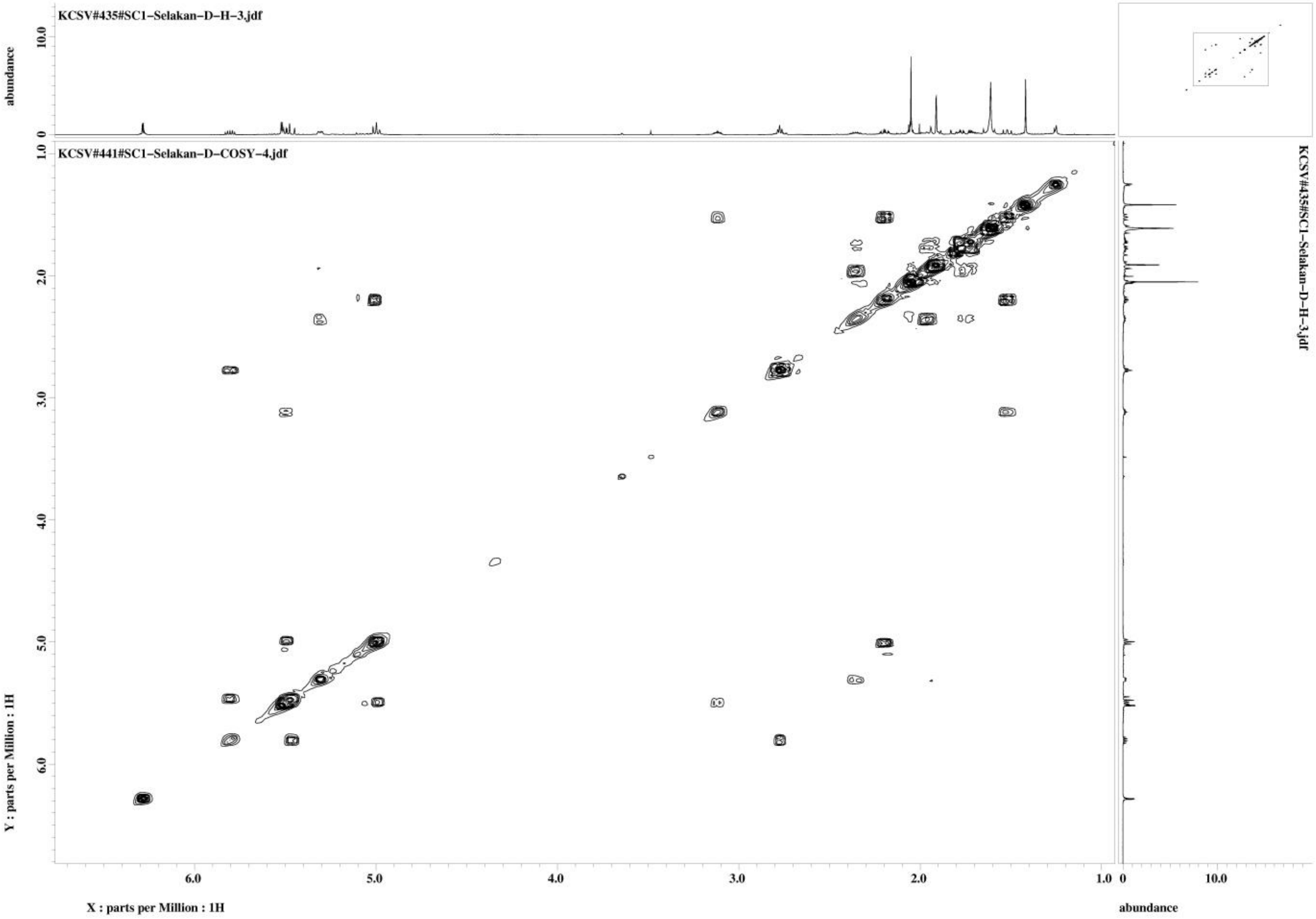

Figure S11. ^{1}H-^{1}H COSY spectrum of **2** in $CDCl_3$.

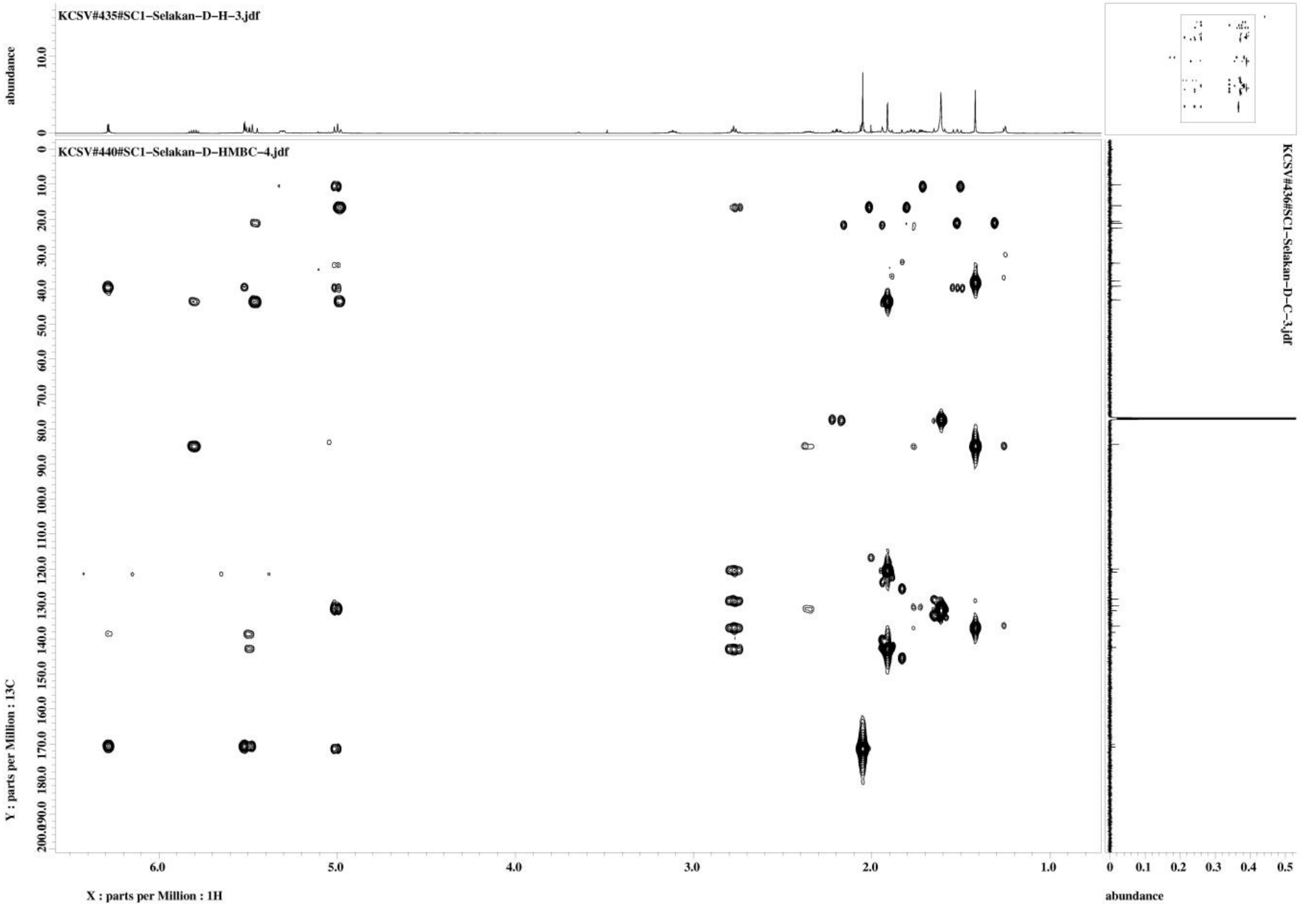

Figure S12. HMBC spectrum of **2** in $CDCl_3$.

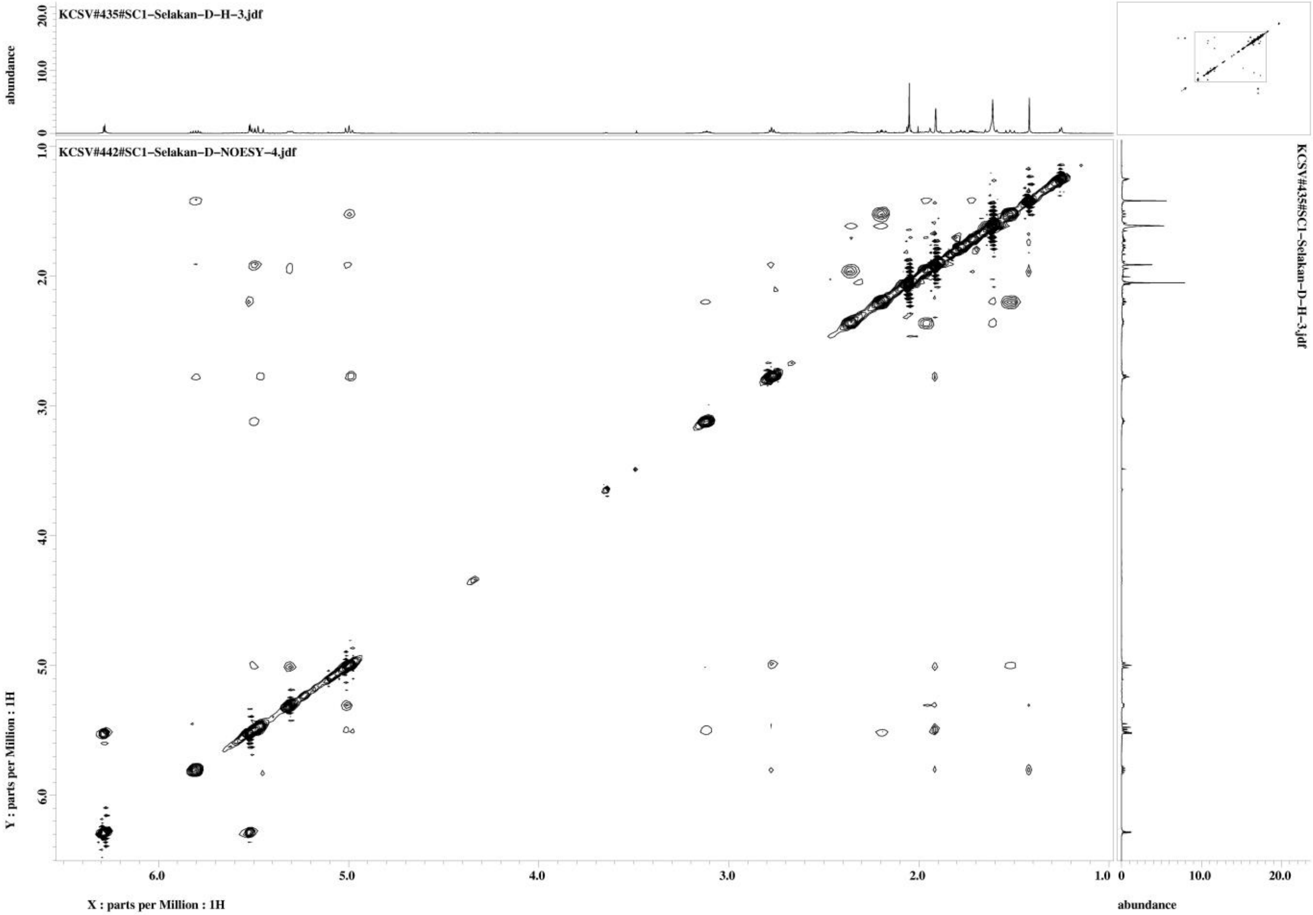

Figure S13. NOESY spectrum of **2** in $CDCl_3$.

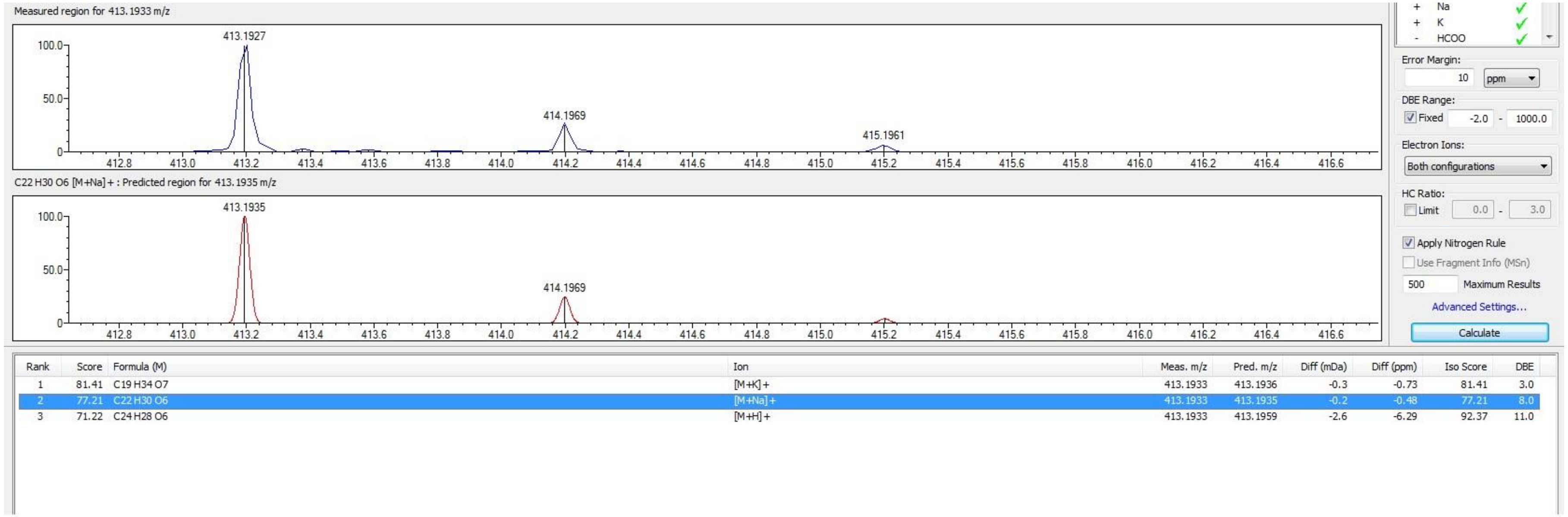

Figure S14. HRESI-MS data of **2**.
